# Key Genes Associated With Functional Specialization of Neonatal Peripheral Monocytes

**DOI:** 10.1155/humu/3009253

**Published:** 2025-08-19

**Authors:** Tingyan Xie, Zicheng Huang, Xian Chen, Zhenchao Jin, Bing Yang, Quan Tang

**Affiliations:** ^1^Research Laboratory, Shenzhen Baoan Women's and Children's Hospital, Shenzhen, Guangdong, China; ^2^Department of Laboratory Medicine, Shenzhen Baoan Women's and Children's Hospital, Shenzhen, Guangdong, China; ^3^Department of Neonatology, Shenzhen Baoan Women's and Children's Hospital, Shenzhen, Guangdong, China; ^4^Department of Cell Biology, College of Basic Medical Sciences, Tianjin Medical University, Tianjin, China; ^5^Department of Public Health, International School, Krirk University, Bangkok, Thailand

**Keywords:** ATAC-seq, monocytes, neonates, peripheral blood, RNA-seq

## Abstract

**Purpose:** The purpose of this study is to identify genes and transcription factors underlying functional differences in neonatal versus adult peripheral blood monocytes, elucidating mechanisms of severe Group B streptococcus (GBS) infection in neonates.

**Methods:** Differentially expressed genes (DEGs) in neonatal and adult peripheral blood monocytes were detected via RNA sequencing (RNA-seq), followed by assay for transposase-accessible chromatin sequencing (ATAC-seq) to characterize differentially accessible region (DAR)–associated genes. Integrated analyses of RNA-seq and ATAC-seq pinpointed candidate genes and transcription factors. Quantitative reverse transcription polymerase chain reaction (qRT-PCR) validated the mRNA expression of common genes and transcription factors.

**Results:** RNA-seq profiling of neonatal and adult peripheral monocytes identified 669 overexpressed and 440 underexpressed genes in neonates, with overexpressed genes enriched in bacterial response pathways and underexpressed genes in cytokine production and cell killing pathways. Chromatin accessibility analysis revealed 36,782 differential peaks (21,192 gained, 15,590 lost) in neonatal peripheral monocytes. Integrated RNA-seq and ATAC-seq analysis pinpointed 30 overlapping genes among DEGs, DAR-associated genes, and immunologically relevant genes (IRGs). qRT-PCR validated higher expression of *CEBPB*, *JUN*, *BATF*, *PTK2B*, and *ITGAV* and lower *ADA2* and *RORA* expression in neonatal peripheral monocytes compared to that in adults.

**Conclusions:** The study revealed distinct differences in the transcriptome and chromatin accessibility between neonatal and adult peripheral monocytes, identifying potential genes linked to GBS infection vulnerability of neonates. These findings advance our understanding of neonatal immune dysfunction in severe GBS disease, informing future therapeutic targets.

## 1. Introduction

Group B streptococcus (GBS) represents a primary drive of serious infections with adverse outcomes in infants [1]. Globally, neonatal GBS infection has an occurrence rate of 0.49‰, accompanied by a mortality rate of 8.4% [2]. Currently, the principal clinical strategies to mitigate neonatal GBS infection are maternal screening and intrapartum antibiotic prophylaxis (IAP) [3]. Although IAP has proven effective in decreasing early neonatal GBS infection, its outcome on late neonatal GBS infection remains limited [4]. Additionally, survivors often grapple with significant sequelae [5], emphasizing the urgency of targeting prevention and treatment.

The underlying mechanisms of severe GBS infection in neonates remain elusive, which poses a challenge in developing novel prevention and treatment. Approximately 30% of healthy adults harbor symbiotic GBS in their gastrointestinal and reproductive tracts; however, it can trigger severe infectious disease and even death in newborns [6, 7]. Neonates mainly rely on the innate immune system to defend against bacteria [8]. Monocytes, key components of the innate immune system, not only phagocytose and kill bacteria directly but also present antigens to T cells indirectly. Unraveling the differences in monocytes between newborns and adults could shed light on the mechanisms underlying severe GBS infections in neonates.

Throughout this study, transcriptional and chromatin accessibility profiles between neonatal and adult peripheral blood monocytes were compared via integrated RNA sequencing (RNA-seq) and assay for transposase-accessible chromatin sequencing (ATAC-seq) analyses, identifying potential genes and transcription factors underlying functional disparities in neonatal monocytes. These findings provide insights into mechanisms associated with severe GBS infection in neonates.

## 2. Materials and Methods

### 2.1. Subjects and Cell Isolation

Eligible 68 neonates (30–41 weeks of gestation) delivered at the Shenzhen Baoan Women's and Children's Hospital and 65 control adults (aged 21–55 year) were included. Neonates with congenital malformations, genetic abnormalities, chromosomal disorders, inborn errors, severe congenital immune disorders, and elevated infection parameters (C-reactive protein) were excluded from the study. The study was approved by the Ethics Committee of Shenzhen Baoan Women's and Children's Hospital (Approval Number LLSC-2021-04-01-05-KS), and all participants or their legal guardians provided written informed consent prior to enrollment. This study was performed in compliance with the Declaration of Helsinki.

Peripheral blood mononuclear cells (PBMCs) were extracted from fresh peripheral blood samples of neonates and adults by using density gradient centrifugation with Ficoll (Cytiva, Uppsala, Sweden). CD14^+^ monocytes were sorted from PBMCs using CD14 MicroBeads (Miltenyi Biotec, Bergisch Gladbach, North Rhine-Westphalia, Germany), with purity typically exceeding 95%.

### 2.2. RNA-Seq Library Construction and Data Analysis

TRIzol (Invitrogen, Carlsbad, California, United States) was used to isolate total RNA from CD14^+^ monocytes in peripheral blood samples of six neonates and four adults. Following purification and quantification, RNA-seq libraries were generated using the NEBNext Ultra RNA Library Prep Kit for Illumina (Catalog E7530L, NEB, Ipswich, Massachusetts, United States). Library sequencing was performed on an Illumina NovaSeq 6000 (IGE Biotechnology Co. Ltd., Guangzhou, Guangdong, China) [9].

Sequencing reads were initially subjected to quality control using FastQC (Version 0.11.9) and were subsequently trimmed with Trim Galore (Version 0.6.10) to eliminate adapters and low-quality reads. HISAT2 (Version 2.2.1) mapped the clean reads to the hg19 reference genome. Gene-level transcript quantification from mapped reads was performed using featureCounts (Version 2.0.1) with GENECODE annotations. Fragments per kilobase million mapped reads (FPKM) values were calculated using the StringTie software (Version 2.1.7). The ComBat-seq batch adjustment algorithm within the SVA R Package (Version 3.50.0) was employed to correct batch effects. The DESeq2 R package (Version 1.42.1) performed differential expression analysis on RNA-seq data. Differential gene expression between neonatal and adult peripheral monocytes was identified using a threshold of |log2 fold change| > 1 and adjusted *p* value < 0.05. The ClusterProfiler R package (Version 4.10.1) performed gene ontology (GO) enrichment analysis, designating GO terms with *p* < 0.05 as significantly enriched.

The Gene Set Enrichment Analysis (GSEA) software (http://www.gsea-msigdb.org, Version 4.1.0) was employed to conduct GSEA, with statistical significance defined by absolute normalized enrichment score (NES) > 1, *p* < 0.05, and false discovery rate (FDR) < 0.25.

### 2.3. ATAC-Seq Library Construction and Data Analysis

The CD14^+^ monocytes of three neonates and three adults were included to construct the ATAC-seq library. Briefly, 50,000 cells were treated with cold lysis buffer and pelleted by centrifugation to isolate nuclei, which were redispersed in the transposase reaction buffer and incubated with shaking at 300 rpm for 30 min at 37°C. After purification of the transposed DNA, libraries were generated via 10 cycles of amplification using the KAPA HiFi HotStart ReadyMix Kit (Roche, Basel, Switzerland). Following quality control and quantification, libraries underwent sequencing using the Illumina system [10, 11].

FastQC (Version 0.11.9) initiated quality control of sequencing reads, with adapter and low-quality sequence removal subsequently performed via Trim Galore (Version 0.6.10). Bowtie2 (Version 2.4.2) aligned filtered reads to the reference genome hg19 using parameters --very-sensitive -X 2000, followed by the removal of duplicated reads and mitochondrial sequences. MACS2 (Version 2.2.7.1) executed peak calling with parameters -nomodel -shift -100 -extsize 200 -B -p 0.05.

DeepTools (Version 3.5.1) was employed to perform counts per million mapped reads (CPM) normalization, generating a big-wig file that can be visualized in the Integrated Genomic Viewer (IGV, Version 2.17.3).

The block parameter in dba.contrast function allowed for batch effects by including batch information in DiffBind (Version 3.12.0). DiffBind identified differential peaks with |log2 fold change| > 1 and *p* < 0.05, which were annotated via the ChIPseeker package (Version 1.38.0).

### 2.4. Integrative Analyses of RNA-Seq and ATAC-Seq

Immunologically relevant gene (IRG) inventory (Supporting Information 3: Table S1) was acquired from the Immunology Database and Analysis Portal (ImmPort, https://www.immport.org/home). After removing redundant genes, a total of 1509 IRGs were obtained. The common genes of differentially expressed genes (DEGs) and differentially accessible region (DAR)–associated genes were input to ImmPort to identify immunologically associated genes. The workflow of the integrative analysis is shown in Supporting Information 1: Figure S1.

The raw data of GSE60216 (single-end sequencing data of neonatal cord blood monocytes and adult peripheral blood monocytes) [12] was downloaded from the GEO database. Raw reads underwent quality control. HISAT2 (Version 2.2.1) mapped clean reads to the hg19 reference genome. Gene expression levels were quantified by featureCounts (Version 2.0.1) and normalized by the FPKM method. A bar chart depicted the FPKM values (mean ± SEM) of the common genes in monocytes between cord blood in neonates and peripheral blood in adults.

### 2.5. Motif Analysis

Motif analysis via the findMotifsGenome.pl script from the HOMER suite (Version 4.11) characterized enriched motifs in peak-associated genomic regions of target genes. The default parameters of findMotifsGenome.pl were employed, along with the -find option to identify motifs matching the peaks.

### 2.6. RNA Preparation and qRT-PCR

The EZ-press RNA Purification Kit (EZBioscience, Roseville, Minnesota, United States) was employed for cell lysis and extraction of total cellular RNA. The BioZues III First Strand cDNA Synthesis Kit (BiOligo, Shanghai, China) was utilized for cDNA synthesis. qRT-PCR was performed using the GoTaq qPCR Master Mix (Promega, Madison, Wisconsin, United States). Data were calibrated using the 2^−ΔΔCT^ normalization method with *β-actin* as the reference gene, and primer sequences are detailed in Table 1.

### 2.7. Prediction of Binding Sites for Transcription Factors and Target Genes

The promoter sequences (from −2000 bp to transcription start site [TSS]) of target genes were retrieved from the NCBI nucleotide database. The latest transcription factor binding motifs from the JASPAR database (https://jaspar.elixir.no/, Version 2024) were used to scan promoter sequences with a relative profile score threshold of 0.8, identifying potential transcription factor binding sites. Higher scores indicated greater prediction confidence, with the highest scoring site designated as the most probable binding site. Additionally, transcription factor motifs (MEME format) and promoter sequences were analyzed via the MEME/MAST suite (https://meme-suite.org/meme/tools/mast, Version 5.5.7) for motif scanning, with identified matches filtered to confirm their presence in promoter regions.

### 2.8. Statistical Analysis

Mean ± SD was used to present qRT-PCR data. When comparing two independent samples, homogeneity of variance was assessed using the *F*-test. Two-tailed *t*-tests or Welch's *t*-tests were applied depending on whether variances were homogeneous or not. Statistical significance was assigned to differences with a *p* value < 0.05.

## 3. Results

### 3.1. RNA-Seq Analysis Showed a Different Gene Profile in Neonatal and Adult Peripheral Monocytes

**Figure 1:**
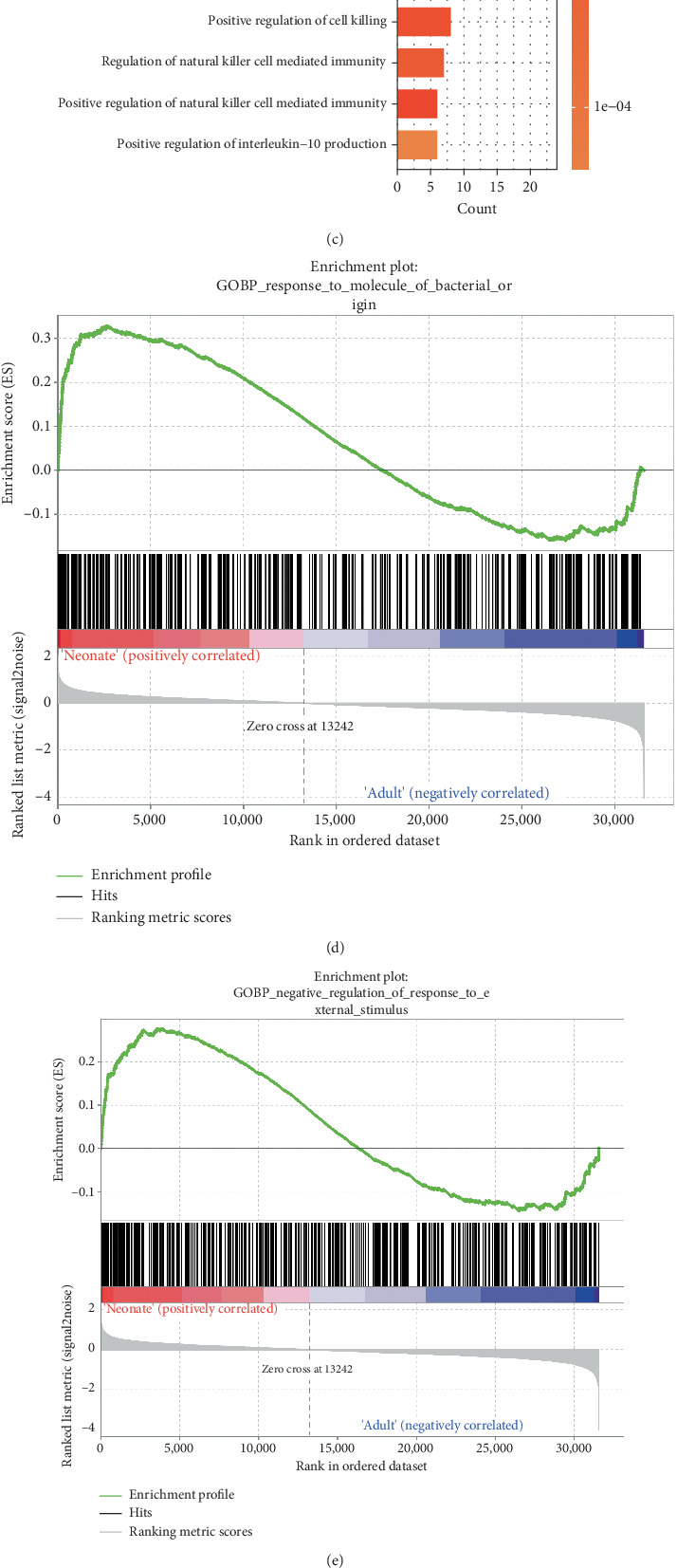
Analysis of differential gene expression and functional characterization between neonatal and adult peripheral monocytes. (a) Volcano plot of DEGs between neonatal and adult peripheral monocytes. Blue dots: underexpressed DEGs, red dots: overexpressed DEGs, gray dots: genes with no significant changes; adjusted *p* < 0.05, |log2 FC > 1|. Enriched GO biological process terms for (b) Top 10 overexpressed and (c) Top 10 underexpressed DEGs in neonatal peripheral monocytes. GSEA shows the upregulation of (d) response to molecule of bacterial origin and (e) negative regulation of response to external stimulus and the downregulation of (f) positive regulation of cytokine production and (g) regulation of cell killing in neonatal peripheral monocytes. DEGs: differentially expressed genes; GSEA: Gene Set Enrichment Analysis.

The different gene expression profile of peripheral monocytes of neonates and adults was analyzed through RNA-seq. In neonatal peripheral monocytes, 1109 DEGs were determined, including 669 overexpressed and 440 underexpressed genes (Figure 1a and Supporting Information 4: Table S2). GO biological process analysis was performed to further characterize the functional roles of the identified DEGs.

The overexpressed genes were predominantly clustered in response to bacteria-associated pathways (Figure 1b), while the underexpressed genes primarily focused on cytokine production and cell killing pathways (Figure 1c). GSEA further confirmed the upregulation of bacteria-associated pathways and the downregulation of cytokine production and cell killing pathways in neonatal peripheral monocytes (Figures 1d, 1e, 1f, and 1g and Supporting Information 2: Figure S2).

### 3.2. ATAC-Seq Analysis Showed a Different Chromatin Accessibility Landscape in Neonatal and Adult Peripheral Monocytes

**Figure 2:**
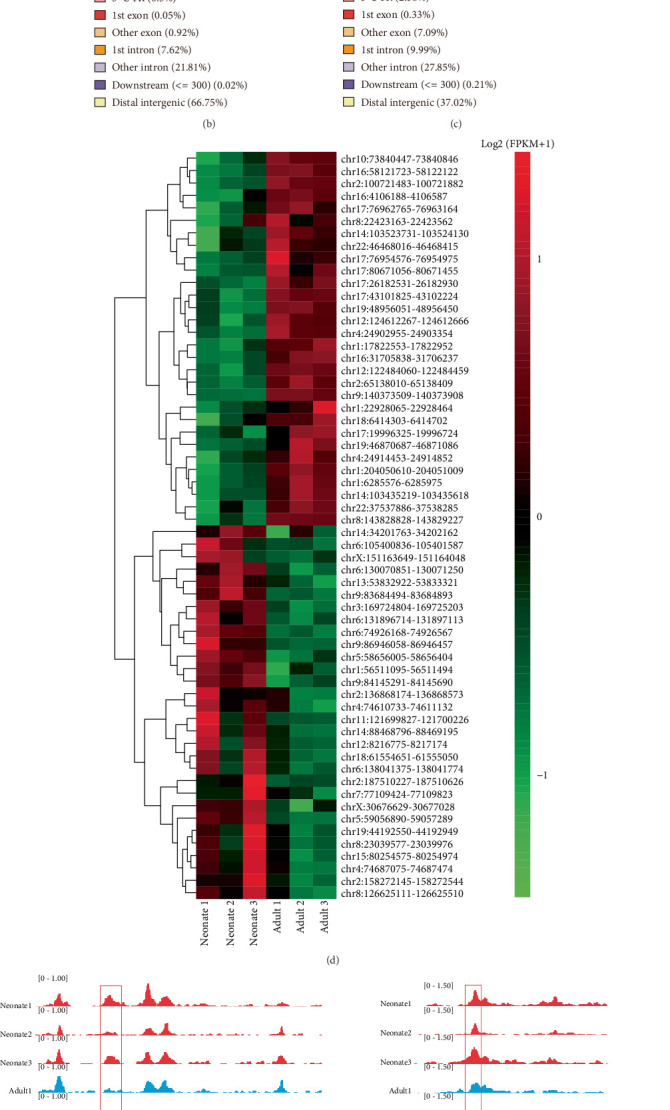
Comparative chromatin accessibility profiling of neonatal and adult peripheral monocytes. (a) Differential chromatin accessibility analysis identifies DARs in neonatal peripheral monocytes. Red plots denote differentially accessible regions (*p* < 0.05, |log2 FC| > 1), with dots above the line indicating gained peaks and below indicating lost peaks in neonatal peripheral monocytes. Distinct distributions of DARs in different genomic regions in (b) neonatal and (c) adult peripheral monocytes. (d) Heatmap visualizes the Top 30 overexpressed and Top 30 underexpressed differentially accessible regions in neonatal peripheral monocytes. (e) IGV shows differences in DAR signals (highlight in red rectangles) of *GPR65*, *CXCL8*, *DCAKD*, and *CDC42BPB* genes between neonatal (red) and adult (blue) peripheral monocytes.

Chromatin accessibility differences between neonatal and adult peripheral monocytes were analyzed using ATAC-seq. All peaks are shown by scatter plot in Figure 2a.

The differential chromatin accessibility analysis identified a total of 36,782 peaks between neonatal and adult peripheral monocytes, including 21,192 peaks gained and 15,590 peaks lost (*p* < 0.05, |log2 FC| > 1) in neonatal peripheral monocytes (Figure 2a).

Pie diagrams illustrate the different distributions of differential chromatin accessibility in neonatal and adult peripheral monocytes. In the neonatal peripheral monocytes, 66.75% of gained peaks are located in distal intergenic, 21.81% in other introns, and 7.62% in the first intron (Figure 2b). In contrast, in the adult peripheral monocytes, 37.02% of lost peaks are located in distal intergenic, 27.85% in other introns, 9.99% in the first intron, and 7.09% in other exons (Figure 2c). Interestingly, only 2.42% of gained peaks are located in the promoter (including ≤ 1, 1–2, and 2–3 kb) region in neonatal peripheral monocytes, while 14.45% of lost peaks are located in the promoter region (including ≤ 1, 1–2, and 2–3 kb) adult peripheral monocytes. The 281 genes overlapping between RNA-seq and ATAC-seq analyses were identified, whose DARs are concluded in Supporting Information 5: Table S3. Among these genes, the DARs of the Top 30 overexpressed and Top 30 underexpressed in neonatal peripheral monocytes are shown in the heatmap (pheatmap package v1.0.12), with a significant difference (Figure 2d). For example, IGV shows that the chromatin accessibility levels of G protein-coupled receptor 65 (*GPR65*) and C-X-C motif chemokine ligand 8 (*CXCL8*) are elevated in neonatal peripheral monocytes, while dephospho-CoA kinase domain containing (*DCAKD*) and CDC42 binding protein kinase beta (*CDC42BPB*) are lower (Figure 2e).

### 3.3. Integrative Analyses of RNA-Seq and ATAC-Seq

**Figure 3:**
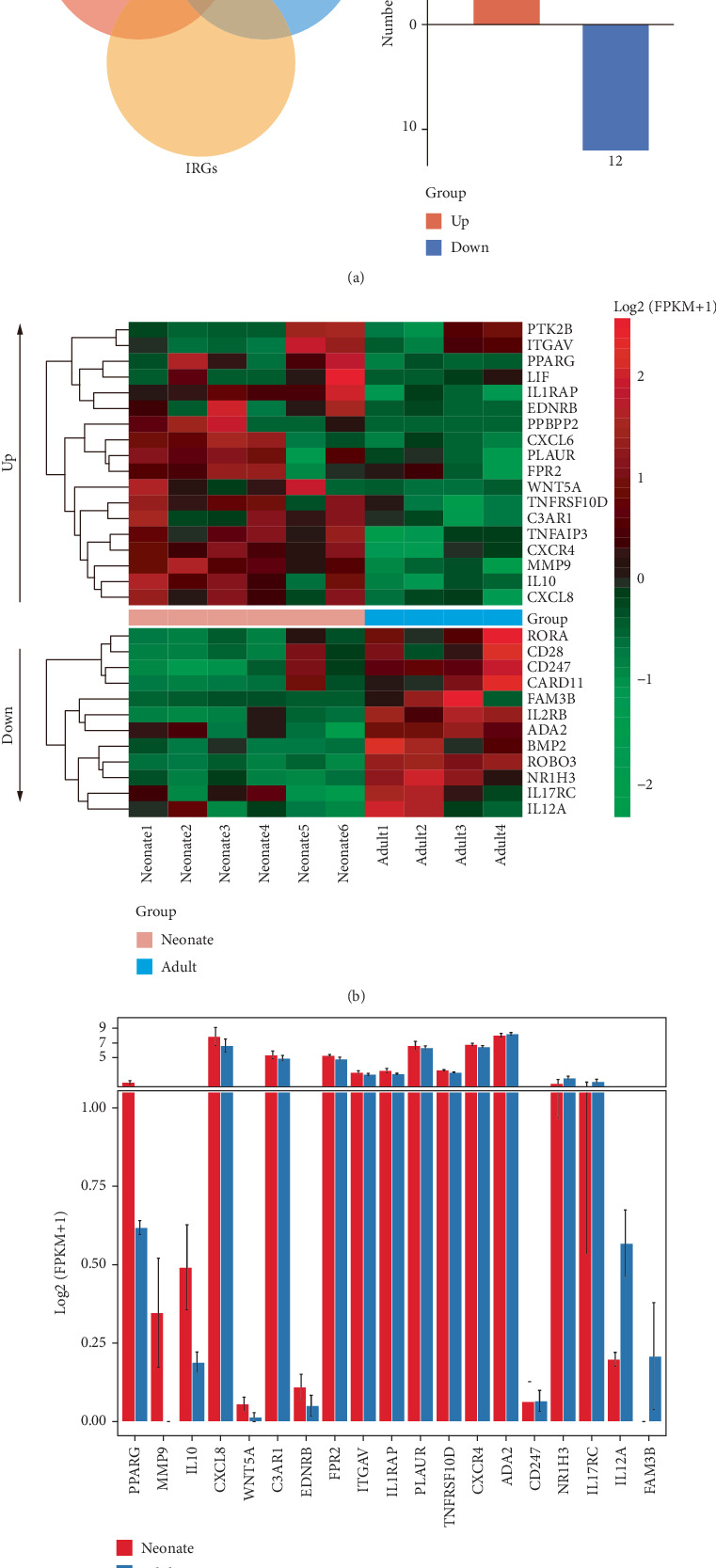
Integrated analysis of DEGs, DAR-associated genes, and IRGs. (a) A Venn diagram illustrates 30 common genes of DEGs, DAR-associated genes, and IRGs, with 18 overexpressed and 12 underexpressed in neonatal peripheral monocytes. (b) Heatmap of the expression levels of the 30 common genes, with color intensity representing relative expression from red (high) to green (low). (c) Bar graph shows the expression levels of 19 common genes identified in the GSE60216 dataset. Red: monocytes in neonatal cord blood, gray: monocytes in adult peripheral blood. Data are presented as mean ± SEM.

Via integration analysis, we found 30 common genes of DEGs, DAR-associated genes, and IRGs from ImmPort, with 18 overexpressed and 12 underexpressed genes in neonatal peripheral monocytes (Figure 3a).

The heatmap visualized the normalized expression of the 30 shared genes (Figure 3b). The expression of the 30 genes was also analyzed in an additional publicly available dataset (GSE60216 dataset). Elevated expression of peroxisome proliferator-activated receptor gamma (*PPARG*), matrix metalloproteinase 9 (*MMP9*), interleukin 10 (*IL10*), *CXCL8*, Wnt family member 5A (*WNT5A*), complement C3a receptor 1 (*C3AR1*), integrin subunit alpha V (*ITGAV*), interleukin 1 receptor accessory protein (*IL1RAP*), formyl peptide receptor 2 (*FPR2*), plasminogen activator, urokinase receptor (*PLAUR*), TNF receptor superfamily member 10d (*TNFRSF10D*), endothelin receptor type B (*EDNRB*), and C-X-C motif chemokine receptor 4 (*CXCR4*) was observed in neonatal cord monocytes versus adult peripheral monocytes, while the expression of adenosine deaminase 2 (*ADA2*), CD247 molecule (*CD247*), nuclear receptor subfamily 1 group H member 3 (*NR1H3*), interleukin 12A (*IL12A*), FAM3 metabolism regulating signaling molecule B (*FAM3B*), and interleukin 17 receptor C (*IL17RC*) was lower than that in adults, showing that most genes are with the same trend as our data (Figure 3c).

### 3.4. Motif Analysis of the Common Gene-Associated Peaks

**Figure 4:**
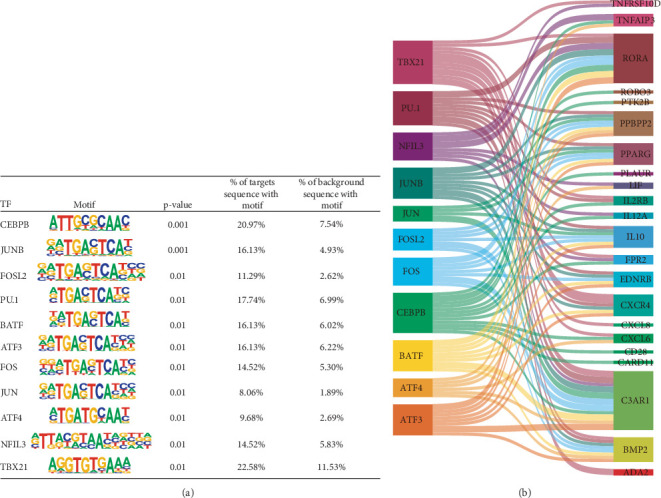
Motif analysis of DARs associated with the common genes. (a) The transcription factors enriched in accessibility regions of the common genes. (b) Sankey diagram of the potential transcriptional regulation network, with transcription factors (left column) linked to target common genes (right column).

Based on motif analysis of the 30 genes associated with peaks, 11 transcription factors were identified, including Fos proto-oncogene (*FOS*), basic leucine zipper ATF-like transcription factor (*BATF*), CCAAT enhancer-binding protein *β* (*CEBPB*), and Jun proto-oncogene (*JUN*) (Figure 4a). Meanwhile, we constructed a Sankey diagram (ggsankey package v0.0.99999), including the 11 transcription factors and 22 common genes, showing the potential transcriptional regulation network (Figure 4b).

### 3.5. qRT-PCR Verification of Common Genes

**Figure 5:**
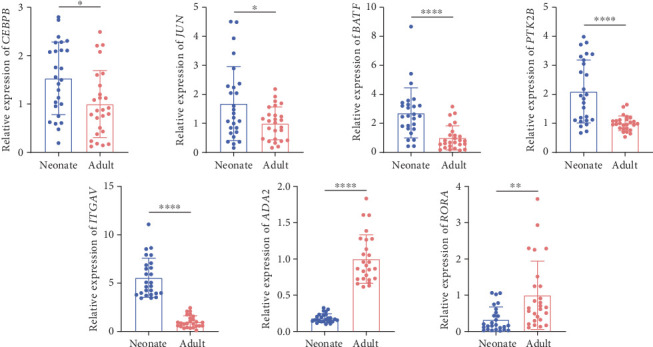
Validation of the common gene via qRT-PCR. The expression of *CEBPB*, *JUN*, *BATF*, *PTK2B*, and *ITGAV* was significantly elevated in neonatal versus adult peripheral monocytes, whereas the expression of *ADA2* and *RORA* was significantly lower. ⁣^∗^*p* < 0.05, ⁣^∗∗^*p* < 0.01, and ⁣^∗∗∗∗^*p* < 0.0001.

To verify the results, the expression of the 30 shared genes and transcription factors was further analyzed via qRT-PCR.  Figure 5 illustrates elevated expression of transcription factors *CEBPB*, *JUN*, *BATF*, protein tyrosine kinase 2*β* (*PTK2B*), and *ITGAV* in neonatal peripheral monocytes relative to adult counterparts, while the expression of *ADA2* and RAR-related orphan receptor A (*RORA*) was significantly lower.

### 3.6. Prediction of Transcription Factor Binding Motifs in Target Gene Promoters

Binding motifs of transcription factors BATF, CEBPB, and JUN in the promoters of *ITGAV*, *PTK2B*, *ADA2*, and *RORA* were computationally identified via the JASPAR database. Predictions showed BATF had potential binding sites across all four genes; CEBPB bound *ADA2* and *RORA* but not *ITGAV* or *PTK2B*; JUN bound *PTK2B*, *ADA2*, and *RORA* but not *ITGAV*. Motif scanning performed by the MEME/MAST suite also predicted the significant matches for both BATF and JUN motifs in the *PTK2B* promoter region, supporting their binding (Table 2).

## 4. Discussion

Neonatal GBS infection often leads to severe disease and mortality, yet GBS typically colonizes adults asymptomatically. Underlying pathogenic mechanisms driving this neonatal susceptibility remain undefined. As innate immune cells critical for bacterial phagocytosis, monocytes are thought to contribute significantly to this disparity. Here, multiomics profiling of peripheral blood monocytes via RNA-seq and ATAC-seq was conducted to characterize immune functional disparities between neonates and adults, dissecting regulatory mechanisms at transcriptional and chromatin accessibility levels.

RNA-seq analysis of neonatal and adult peripheral blood monocytes revealed extensive gene expression differences. Prior studies utilizing microarray or single-end RNA-seq analyzed neonatal cord blood monocytes, constrained by technical limitations: microarray is restricted to predesigned probes for known transcripts, while single-end sequencing lacks the depth to resolve subtle expression changes [12, 13]. In contrast, our paired-end RNA-seq analysis directly profiled peripheral blood monocytes from both neonates and adults, which are rarely reported due to the limited availability and challenging procurement of neonatal peripheral blood samples.

Enrichment analysis revealed neonatal peripheral monocytes DEGs were primarily enriched in response to bacteria-associated pathways, whereas adult peripheral monocyte DEGs clustered in cytokine production and cell-killing pathways. These results highlight distinct immune functional profiles of neonatal versus adult peripheral monocytes. De Jong et al. reported lower neonatal glycolytic capacity underlies immune response disparities with adults, potentially contributing to heightened infection tolerance and sepsis susceptibility in neonates [14]. Compared to adults, neonates exhibit phagocytic impairments, including defective bacterial ingestion [15]. Lissner et al. discovered age-dependent monocyte gene expression differences—such as interferon regulatory factor 3 (IRF3)—as key drivers of neonatal innate immune deficiencies linked to increased infection vulnerability [12].

This study provides the first ATAC-seq characterization of chromatin landscapes in neonatal peripheral blood monocytes. ATAC-seq, which assesses chromatin accessibility, reveals epigenetic landscapes and predicts upstream transcription factors of genes [16]. Our analysis identified distinct chromatin accessibility patterns between neonatal and adult peripheral monocytes, with differentially distributed accessible regions highlighting functional specializations in neonatal peripheral monocytes relative to adult counterparts. Motif analysis of DARs predicted transcription factors potentially driving gene-specific regulatory programs underpinning these functional disparities.

It is the first time to perform RNA-seq and ATAC-seq integrated analyses of monocytes in peripheral blood samples of neonates. Integrating DEGs, DAR-associated genes, and IRGs, we identified 30 candidate regulatory genes. The expression of *PTK2B*, *ITGAV*, *ADA2*, *RORA*, and associated transcription factors *CEBPB*, *JUN*, and *BATF* was validated by qRT-PCR. No prior comparative analysis of gene expression profiles in neonatal versus adult peripheral blood monocytes has been reported.

The expression of *CEBPB*, *JUN*, *BATF*, *PTK2B*, and *ITGAV* was significantly higher in neonatal peripheral monocytes than that in adults. CEBPB, a member of the CCAAT enhancer binding protein family, binds DNA as a homodimer or heterodimer to regulate immune and inflammatory response genes [17]. In French–American–British (FAB) acute myelomonocytic/monoblastic and monocytic leukemia cells, a high CEBPB level promotes monocytic differentiation [18]. Evidence suggests that CEBPB deficiency correlates with augmented macrophage phagocytosis [19].

JUN is a member of the Jun transcription factor family [20]. The encoded protein is known as JUN or c-Jun [21]. In Tohoku Hospital Pediatrics-1 (THP-1) monocytic cells stimulated by interleukin 1 beta (IL-1*β*), the absence of c-Jun results in diminished expression of C-X-C motif chemokine ligand 10 (CXCL10) [22]. Under specific conditions, the upregulation of the c-Jun-containing transcription complex impairs phagocytosis through the upregulation of Fc gamma receptors IIb [23].

BATF is a member of the activating protein-1 (AP-1) transcription factor family [24]. BATF is a core transcription factor that leads to impaired natural killer (NK) cell killing capacity in acute myeloid leukemia [25]. BATF deficiency also inhibits the differentiation of monocytes into osteoclasts induced by tumor necrosis factor (TNF) superfamily member 11 (RANKL) [26].

A Ca^2+^-activated nonreceptor tyrosine kinase associated with focal adhesion kinase, Pyk2 is encoded by PTK2B [27]. Inhibition of Pyk2 induces multinucleation of microglia, enhancing phagocytic activity and lysosomal activity [28]. Pyk2 is localized to phagosomes and podosomes, structures critical for phagocytosis. Pyk2 recruitment to these structures is essential for the phagocytic function of alveolar macrophages [29].

ITGAV is an integrin *α* subunit also known as CD51 [30]. Krispin et al. found that CD51 on immature dendritic cells binds thrombospondin-1 (TSP-1) on apoptotic monocytes, enhancing the phagocytic and immunosuppressive functions of immature dendritic cells [31]. In the mouse *Salmonella* infection model, CD51 facilitates nonprofessional efferocytosis, a major antimicrobial mechanism, in neonatal intestinal epithelial cells [32]. In gastric mucosa-infiltrating monocytes, ITGAV indirectly has an effect on bacterial burden via a series of signaling pathways [33].

The expression of *ADA2* and *RORA* in neonatal peripheral monocytes was significantly lower than that in adults. ADA2 encodes a dimeric enzyme belonging to the adenosine deaminase growth factor family, which is primarily produced by monocytes [34], functioning in Toll-like receptor 9 (TLR9) activation in lysosomes to regulate immune sensing of DNA [35]. ADA2 deficiency leads to excessive inflammatory responses in humans. While restoration of ADA2 function normalizes inflammatory activation in macrophages, ADA2-deficient cells excessively produce proinflammatory cytokines interleukin 6 (IL-6) and TNF [36].

RORA, a nuclear hormone receptor, functions by binding the specific ligands to regulate adjacent gene expression [37]. The suppression of proinflammatory macrophage polarization induced by melatonin is mediated by RORA [38]. In nonalcoholic steatohepatitis conditions, RORA induces Kruppel-like factor 4 (KLF4) to promote anti-inflammatory macrophage polarization, thereby ameliorating disease progression [39].

Our data predicted PTK2B as a target of JUN and BATF transcription factors. While Reza et al. reported JUN-mediated regulation of PTK2B using the multiomics approach [40], the transcriptional regulation of PTK2B by BATF represents a previously unreported mechanism. This finding predicted BATF as a novel regulator of PTK2B in neonatal peripheral monocytes, expanding our understanding of molecular interactions underlying neonatal immune responses.

The study revealed distinct transcriptome and chromatin accessibility profiles between neonatal and adult peripheral monocytes, identifying potential genes associated with neonatal vulnerability to severe GBS infection. These findings enhance understanding of immune dysfunction in neonatal severe GBS disease and provide insights for developing preventive and therapeutic targets. The identified genes and transcription factors warrant further experimental validation to elucidate their functional roles in neonatal immune responses.

## Figures and Tables

**Table 1:** Nucleotide sequences of primers used for qRT-PCR detection.

**Primer**	**Sequence (5**⁣′** to 3**⁣′**)**
*β-Actin*	Forward: CATGTACGTTGCTATCCAGGC
	Reverse: CTCCTTAATGTCACGCACGAT
*PTK2B*	Forward: CATCGTGAAGCTGATCGGCATC
	Reverse: TCTTGTTCCGCTCCAGGTAGTG
*ITGAV*	Forward: AGGAGAAGGTGCCTACGAAGCT
	Reverse: GCACAGGAAAGTCTTGCTAAGGC
*ADA2*	Forward: GCAGGAGTTCTACGAGGACAAC
	Reverse: CTTCGTCATGGTGCTCTCCACT
*RORA*	Forward: CACCAGCATCAGGCTTCTTTCC
	Reverse: GTATTGGCAGGTTTCCAGATGCG
*CEBPB*	Forward: AGAAGACCGTGGACAAGCACAG
	Reverse: CTCCAGGACCTTGTGCTGCGT
*JUN*	Forward: CCTTGAAAGCTCAGAACTCGGAG
	Reverse: TGCTGCGTTAGCATGAGTTGGC
*BATF*	Forward: TATTGCCGCCCAGAAGAGC
	Reverse: GCTTGATCTCCTTGCGTAGAG

**Table 2:** Prediction of binding sites for transcription factors and target genes via JASPAR database or MEME/MAST suite.

**Transcription factors**	**Target gene**	**Relative score**	**p** ** value**	**Start**	**End**	**Strand**	**Predicted sequence**
BATF	ITGAV	0.93	NA	173	179	−	TGACTAA
(MA1634.2)	PTK2B	1.00	6.5e − 5	1124	1130	+	TGACTCA
	ADA2	0.88	NA	682	688	+	TGACACA
	RORA	0.89	NA	24	30	+	TGACTTA
CEBPB	ITGAV	NA	NA	NA	NA	NA	NA
(MA0466.4)	PTK2B	NA	NA	NA	NA	NA	NA
	ADA2	0.83	NA	656	665	+	ATTGTGCCAC
	RORA	0.82	NA	336	345	+	ATTGTGCATT
JUN	ITGAV	NA	NA	NA	NA	NA	NA
(MA0489.1)	PTK2B	0.92	NA 4.5e − 5	1123 1123	1136 1136	−	AATGCATGAGTCAG CTGACTCATGCATT
	ADA2	0.85	NA	676	689	+	CCTAGGTGACACAG
	RORA	0.82	NA	102	115	−	ATTGGCTGCCTCAT

*Note:* Relative score was generated by JASPAR database. The numerical range of the relative score is from 0 to 1. The higher the relative score, the more reliable the predicted binding site. The *p* value was generated by MEME/MAST suite.

Abbreviation: NA, not available.

## Data Availability

The data that support the findings of this study are available from the corresponding authors upon reasonable request.

## Supporting Information

Supporting Information 1 Figure S1: The workflow of the integrative analysis. RNA-seq: RNA sequencing; ATAC-seq: assay for transposase-accessible chromatin sequencing; DEGs: differentially expressed genes; DARs: differentially accessible regions; IRGs: immunologically relevant genes.

Supporting Information 2 Figure S2: The result of Gene Set Enrichment Analysis (GSEA) in neonatal and adult peripheral monocytes. GSEA shows upregulation of (A) cellular response to a molecule of bacterial origin and (B) cellular response to biotic stimulus in neonatal monocytes and upregulation of (C) positive regulation of cell killing and (D) positive regulation of interleukin-10 production in adult monocytes.

Supporting Information 3 Table S1: The immunologically relevant genes (IRGs), Immunology Database and Analysis Portal.

Supporting Information 4 Table S2: The differentially expressed genes (DEGs) between neonatal and adult peripheral monocytes.

Supporting Information 5 Table S3: The differentially accessible regions (DARs) of the overlapping genes of DEGs and DAR-associated genes between neonatal and adult peripheral monocytes.
